# A pilot multicenter randomized controlled trial comparing Bankart repair and remplissage with the Latarjet procedure in patients with subcritical bone loss (STABLE): study protocol

**DOI:** 10.1186/s40814-022-00987-4

**Published:** 2022-01-31

**Authors:** Moin Khan, Asheesh Bedi, Ryan Degen, Jon Warner, Mohit Bhandari, on behalf of the STABLE Investigators, Moin Khan, Ryan Degen, Mohit Bhandari, Asheesh Bedi, Jon Warner, Kim Madden, Nazanin Barkhordari, Miriam Garrido Clua, Kelsey Wozny, Jaydeep Moro, Matthew Denkers, Olufemi R. Ayeni, Robert Litchfield, Diane Bryant, Stacey Wanlin, Andrew Firth, Stephanie Horst, Katelyn Inch, Peter Lapner, Katie McIlquham, Montserrat Garcia Portabella, Jorge H. Nuñez, Lledo Batalla, Josep Massons, Patrick Henry, Katrine Milner, Yinmin Ou, Monica Kunz, Alicia Alvares, Saranjan Moganathas, Aarani Chandrasegaram, Etinosa Oliogu, Phumeena Balasuberamaniam, Barbara Gundi, Nithila Sivakumar, Khadija Rashid, Stephanie Lewaniak, Atqiya Fariha, Lavaneyaa Sri, Bashar Alolabi, Carlee Bolton, Xinning “Tiger” Li, Emily Curry, Dana Michlin, Davide Bardana, Ryan Bicknell, Heather Grant, Fiona Howells, Peter MacDonald, Jason Old, Jarret Woodmass, Sheila Mcrae, Brittany Bruinooge, Derek McLennan, Rahne Magnusson, Timothy Leroux, Tamara Wagner, Michaela Kopka, Mark Heard, Greg Buchko, Sarah Kerslake, Rachel M. Frank, Eric McMarty, Andres Barandiaran, Kelly Leach, Kyle Suess, Bruce Miller, John Grant, Bethany Ruffino, Anand Murthi, Shawanna Jackson, Rodrigo de Marinis Acle, Rodrigo Liendo Verdugo, Catalina Vidal Olate, Michel van den Bekerom, Derek van Deurzen, Sigrid Vorrink, Ydo V. Kleinlugtenbelt, I.F. Kodde, Ellie Landman, Hannie Elskamp-Meijerman, Monique Voskamp, Raul Barco, Alfonso Vaquero, Abdul-ilah Hachem, C Ventura-Parellada, JM Mora Guix, F Gamez-Baños

**Affiliations:** McMaster University, Mary Grace Wing, Room G807, 50 Charlton Ave E, Hamilton, ON L8N 4A6 Canada

**Keywords:** Shoulder, Instability, Randomized, Trial, Surgery

## Abstract

**Introduction:**

Anterior dislocations, the most common type of shoulder dislocation, are often complicated by subsequent instability. With recurrent dislocations there often is attrition of the labrum and progressive loss of the anterior bony contour of the glenoid. Treatment options for this pathology involve either soft tissue repair or bony augmentation procedure. The optimal management remains unknown and current clinical practice is highly varied.

**Methods and analysis:**

The Shoulder instability Trial comparing Arthroscopic stabilization Benefits compared with Latarjet procedure Evaluation (STABLE) is an ongoing multi-centre, pilot randomized controlled trial of 82 patients who have been diagnosed with recurrent anterior shoulder instability and subcritical glenoid bone loss. Patients are randomized to either soft tissue repair (Bankart + Remplissage) or bony augmentation (Latarjet procedure). The primary outcome for this pilot is to assess trial feasibility and secondary outcomes include recurrent instability as well as functional outcomes up to two years post-operatively.

**Conclusions:**

This trial will help to identify the optimal treatment for patients with recurrent shoulder instability with a focus on determining which treatment option results in reduced risk of recurrent dislocation and improved patient outcomes. Findings from this trial will guide clinical practice and improve care for patients with shoulder instability.

**Trial registration:**

This study has been registered on http://www.ClinicalTrials.gov with the following identifier: ClinicalTrials.gov Identifier: NCT03585491, registered 13 July 2018, https://www.clinicaltrials.gov/ct2/show/NCT03585491?term=NCT03585491&draw=2&rank=1.

**Ethics and dissemination:**

This study has ethics approval from the McMaster University/Hamilton Health Sciences Research Ethics Board (REB) (approval #4942). Successful completion will significantly impact the global management of patients with recurrent instability. This trial will develop a network of collaboration for future high-quality trials in shoulder instability.

**Supplementary Information:**

The online version contains supplementary material available at 10.1186/s40814-022-00987-4.

## Introduction

The shoulder is the most commonly dislocated joint in the body with a global incidence that ranges from 15.3 to 24.8 per 100,000 people [1–3]. In North America, a sampling of individuals presenting with shoulder dislocations to United States (US) emergency departments identified an overall incidence rate in the US of 23.9 (95% confidence interval, 20.8 to 27.0) per 100,000 person-years and a maximum incidence rate (47.8 [95% confidence interval, 41.0 to 54.5]) occurring in those between the ages of 20 and 29 years [1].

Anterior dislocations, the most common type of shoulder dislocation, are often complicated by subsequent instability, and recurrent dislocation, with reported rates as high as 42%. The affected population is primarily young males [2–4]. Shoulder instability commonly results in pain and negatively impacts quality of life [5]. Shoulder pathology is the third most common cause of musculoskeletal pain, and in the USA, estimated direct costs for the treatment of shoulder pathology account for over $7 billion USD annually [6]. A number of long-term studies have demonstrated an association between the recurrent instability episodes and the risk of degenerative arthritis and over 50% of patients with shoulder instability will go on to require surgical intervention [7–9].

With recurrent dislocations, there may be attrition of the labrum and progressive loss of the anterior bony contour of the glenoid in addition to the capsulolabral detachment [3, 10, 11]. Shoulders with recurrent instability that do not undergo operative management demonstrate a higher incidence of arthropathy in comparison to those which underwent surgical stabilization [9]. In instances where bone loss is not present, the capsulolabral soft tissue is repaired anatomically via open or arthroscopic means, referred to as a Bankart procedure. With increasing recognition of combined posterosuperior humeral head impaction, known as a Hill-Sachs lesion, this procedure has been increasingly coupled with tethering or tenodesis of the infraspinatus tendon into the Hill-Sachs lesion, known as a “remplissage” procedure [12–14]. Instances of significant bone loss (> 25%) are commonly treated with a non-anatomic reconstruction involving a bone transfer known as a “Latarjet” coracoid transfer procedure [15].

However, there is controversy regarding the optimal treatment of patients with a mild degree of bone loss (10–20%) [16]. Mild glenoid bone loss combined with humeral head defects is very common with reported rates ranging from 49 to 86% in cases of recurrent instability. It is often unrecognized and underestimated resulting in patients potentially being inadequately treated [17–20].

The more common arthroscopic capsulolabral procedures have strong advocates who argue that with modern techniques, arthroscopic management has acceptably low recurrence rates and restores the anatomy of the shoulder joint. Surgeons who prefer the Latarjet procedure cite the unacceptably high failure rates with soft-tissue stabilization [21]. Although retrospective clinical studies have suggested a reduced recurrence rate with the Latarjet procedure, there is a higher reported complication rate and potential morbidity associated with the open procedure [22, 23].

No comparative randomized control trial has been completed evaluating Bankart repair with concomitant remplissage in comparison to a Latarjet procedure in the setting of mild to moderate bone loss. Meta-analysis of available observational studies comparing arthroscopic Bankart repair and open Latarjet procedures suggests an increased risk of recurrent instability with Bankart repair (21.1%) in comparison to Latarjet (11.6%), Risk ratio (RR) = 1.97 (95% Confidence Interval (CI) 1.32–2.95) Additionally, an increased risk of re-dislocation was identified with 9.5% in those undergoing Bankart repair in comparison to Latarjet (5.0%), RR 1.87 (95% CI 1.04–3.34). Despite this, no significant differences in revision surgery rates for instability or rates of complications between patients treated with either procedure were identified [24]. A prospective trial is needed to identify the optimal treatment for patients with recurrent dislocations. A pilot study is needed prior to a large trial to determine the feasibility of a larger trial in terms of ability to recruit across clinical sites, adherence to study protocol, and ability to follow participants for 24 months.

## Objectives

Prior to a large trial, we will conduct a pilot trial comparing arthroscopic capsuloligamentous repair vs. coracoid transfer (Latarjet procedure) evaluating recurrent dislocation rates and functional outcomes over a 24-month period. The feasibility objectives are to evaluate (1) our ability to recruit patients across clinical sites, (2) our adherence to the study protocol, and (3) our ability to follow patients to 24 months.

Clinical objectives for the pilot trial are exploratory only. These will be the objectives of the definitive trial. We will compare arthroscopic capsuloligamentous repair (Bankart repair + Remplissage) vs. coracoid transfer (Latarjet procedure) on recurrent shoulder dislocations and symptoms of instability up to 24 months post-surgery; shoulder function; health-related quality of life; physical examination: range of motion, strength, stability, return to previous level of activity; and shoulder-related complications and serious adverse events.

## Methods

This trial was prospectively registered with clinicaltrials.gov before the first participant was enrolled (NCT03585491). This protocol is reported according to Standard Protocol Items: Recommendations for Interventional Trials (SPIRIT) reporting guidelines and the pilot results paper will follow the pilot study extension to the Consolidated Standards of Reporting Trials (CONSORT) guidelines.

### Design overview

**Figure 1:**
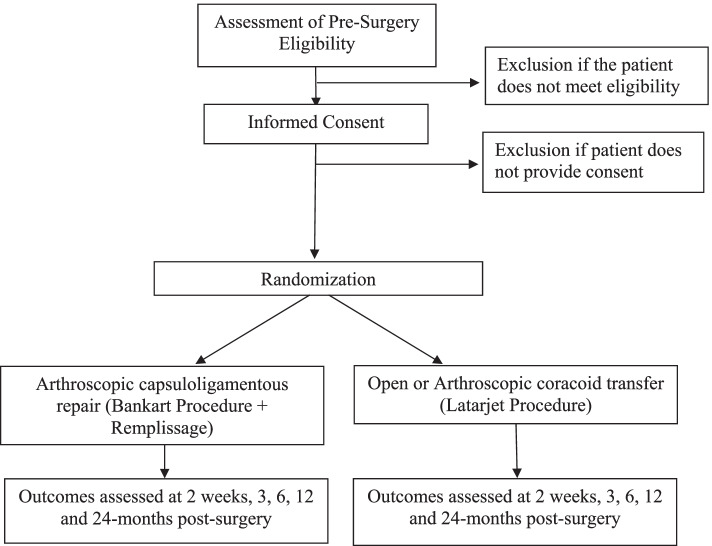
Trial design overview

We propose a multi-centre pilot parallel-group randomized controlled trial of 82 patients across Canada, the USA, and Europe to compare the effect of capsuloligamentous repair (Bankart procedure+ Remplissage) and coracoid transfer (Latarjet procedure) in patients with post-traumatic recurrent anterior dislocation. We will follow eligible and consenting participants for 24 months. We will assess clinical outcomes at 2 weeks, 3 months, 6 months, 12 months, and 24 months post-surgery (Figure 1).

### Study setting

This pilot trial will be conducted at a number of clinical sites in Canada, the USA, and Europe. This study will be coordinated at McMaster University by the Centre for Evidence-Based Orthopaedics (CEO) who will be responsible for trial oversight, clinical site management, data management, data analysis, and knowledge dissemination.

### Eligibility criteria

Patients who meet the eligibility criteria outlined below are to be included in the STABLE study.

The inclusion criteria are:

1. Men and women ages 18–50 years.
2. Diagnosis of post-traumatic recurrent anterior dislocation. This will require a minimum of two episodes of shoulder dislocations with confirming physical examination eliciting unwanted glenohumeral translation with reproduction of symptoms.
3. Mild glenoid bone loss as defined on computed tomography (CT) by standardized and reproducible best-fit circle technique. The circle will be drawn using an on-face 3D reconstruction of the glenoid approximating a normal glenoid. Patients will be included if the missing anterior glenoid is greater than 10% but is less than 20%.
4. Provision of informed consent.

The exclusion criteria are:

1. Patients with concomitant injuries (cuff tear).
2. Previous shoulder surgery.
3. Patients that will likely have problems, in the judgment of the investigators, with maintaining follow-up.
4. Epilepsy.
5. Patients who are or at risk of being incarcerated.
6. Diagnosis of multidirectional instability.
7. Cases involving litigation or workplace insurance claims [e.g. Workplace Safety and Insurance Board (WSIB)].
8. Confirmed connective tissue disorder (Ehlers-Danlos, Marfans) or Beighton hypermobility score > 6.
9. Pregnancy.

### Recruitment strategy and patient screening

Participating centres will identify patients with recurrent dislocation through outpatient clinics. The surgeon, designated fellow, or resident will conduct a history and physical examination. Patients who are potentially eligible based on history and examination will be invited to participate in the trial. If they agree, written informed consent will be obtained in accordance with Good Clinical Practice and local ethics procedures.

### Trial interventions

Participants will be randomly assigned to undergo arthroscopic capsuloligamentous repair (Bankart repair + Remplissage) or open or arthroscopic coracoid transfer (Latarjet procedure).

### Soft tissue stabilization (control)

For arthroscopic soft tissue repair, the participant will be placed in the lateral decubitus or beach chair position. Standard diagnostic arthroscopy will be performed. The anterior capsulolabral complex will be freed from the anterior aspect of the scapular neck. The anterior aspect of the scapular neck will be decorticated. A capsuloligamentous repair will be performed with the capsule shifted from inferior to superior and repaired on the glenoid face. The number of anchors used for the repair will be left to the discretion of the surgeon. The infraspinatus will be tenodesed into the Hill Sachs defect. Patients will be given a sling for 4 weeks, and participation in sports will not be allowed for 6 months.

### Arthroscopic or open Latarjet procedure (intervention)

The Latarjet procedure will be performed through either open or arthroscopic means left up to the discretion of the operating surgeon. With an open procedure, a deltopectoral approach will be performed, the coracoid will be osteotomized at its base, and the posterior surface flattened. The subscapularis muscle will be split longitudinally with a vertical capsule incision close to the glenoid rim. The coracoid will be positioned flush or slightly medial to the glenoid face and secured in a method up to the discretion of the surgeon. Patients will wear a sling for 4 weeks and undergo an identical standardized rehabilitation protocol to those in the control group. In patients who undergo arthroscopic Latarjet procedure, the procedure will be performed via minimally invasive arthroscopic portals.

### Co-interventions

All participants will follow a standardized 6-phase rehabilitation protocol following surgical intervention (Appendix A). All other peri-operative and post-operative interventions will be left to the discretion of the treating surgeon per local standard care.

### Minimizing expertise bias

Differential expertise bias will be limited by ensuring that all participating surgeons are fellowship trained in shoulder surgery and have performed a sufficient number of cases to limit the potential for expertise bias. Based on the available literature, a minimum of 50 cases of arthroscopic Bankart repair would be required for surgeons to meet our participation requirements [25]. Our participation requirement for surgeons performing open or arthroscopic Latarjet procedures would be experience with at least 20 cases as suggested by the literature to maintain the required proficiency [26–29].

### Randomization

To ensure concealed allocation, eligible patients will be randomized using a centralized online randomization system within the Research Electronic Data Capture (REDCap) system. We will use an allocation ratio of 1:1. We will use random block sizes of 4 and 6, and we will stratify by clinical site. A data manager generated the randomization sequence and is the only team member with access to the sequence. Site research personnel will randomize patients.

### Blinding

The study team including the treating surgeon and study coordinator cannot be blinded as they will be performing the procedure and/or will have access to post-operative imaging and clinical notes. Participants will not be blinded to procedure type given the incision with open coracoid transfer and arthroscopic capsuloligamentous repair are distinctive. However, the data analyst will be blinded to the treatment group. For the definitive trial, if one treatment group shows superiority, the trial steering committee will write two interpretation documents before they know which treatment is superior, one for superiority of Latarjet and one for superiority of Bankart repair. This will minimize interpretation bias [30].

## Outcomes

### Primary outcome: Feasibility

The primary outcome of the pilot study will be feasibility. Specifically, this will include (1) *Recruitment* (recruitment of 82 participants); (2) *Protocol adherence* (number of crossovers); and (3) *Follow-up* (proportion of participants followed at two years). The success of the pilot study will be based upon the following a priori thresholds: (1) Establish an average recruitment rate of 0.5 patients per month at each of 10 active sites, (2) 3 or fewer crossovers across the 82 enrolled patients, and (3) 70 of 82 participants (85%) achieving complete follow-up at 2 years.

### Secondary outcomes: Clinical outcomes

The secondary outcomes are exploratory for this pilot trial. The secondary (clinical) outcomes are:

#### Recurrent dislocation and symptomatic instability

This outcome is critically patient important and is objectively documented in the case of shoulder dislocation or in the case of recurrent symptomatic instability will be patient reported at follow-up.

#### Shoulder function

Measured by Western Ontario Shoulder Instability (WOSI) Index, American Shoulder and Elbow Society (ASES) score, and Shoulder Activity Scale. The WOSI is a self-administered quality of life outcome measure designed for clinical trials evaluating treatments for patients with shoulder instability. It has been shown to have high reliability, validity, and responsiveness [31]. The WOSI score is commonly utilized and has been shown to provide excellent ability to detect variability in severity of post-operative instability symptoms including following shoulder stabilization procedures [32]. The ASES score is designed to assess shoulder function including instability [33]. It allows for patient self-evaluation through 11 items that can be used to generate a score, divided into 2 areas: pain (1 item) and function (10 items).

#### Health-related quality of life

Measured using the EuroQol-5 Dimensions (EQ-5D).

#### Physical examination

Physical examination following surgery will be performed by the operating surgeon and will consist of functional assessment important to patients. Range of motion and strength as well as assessment of shoulder stability are commonly reported outcome measures in the literature when assessing success following shoulder instability surgery [5, 24]. Range of motion will be assessed in forward flexion, abduction, external rotation, and internal rotation. Stability will be assessed primarily via the apprehension-relocation physical examination maneuver which has demonstrated the highest sensitivity in the literature for the diagnosis of anterior instability [34].

#### Return to previous level of activity and sport

The majority of shoulder instability affects young individuals involved in athletic activities and sport. An important aspect in the success of surgical intervention is to return patients back to previous and desired level of activity [35]. This outcome will be patient-reported.

#### Shoulder-related complications and serious adverse events

Major complications will include symptomatic non-union of transferred bone block, hardware penetration into the joint, neurological or vascular injury, or deep vein thrombosis. Adverse events will be classified as serious or non-serious. All serious adverse events will be reported to the Methods Centre.

### Data collection and participant follow-up

The number of patients approached, who are potentially eligible, who agree to participate, and who decline participation (with their reason for refusal) will be recorded. Once participants have provided informed consent, baseline demographics, relevant medical history, and details regarding their diagnosis will be collected from the participant, the attending surgeon, their medical record, and through physical examination. Participants will also complete the patient-reported outcome measures (PROMs) at the time of enrolment. After surgery, surgical and peri-operative details will be collected from the attending surgeon and the participant’s medical records. Adverse events occurring during the surgical procedure or perioperative period will also be documented. Post-operative radiographs will be ordered as standard of care. We will use a centralized REDCap system for all sites.

### Follow-up visits

Participants will be followed for 24 months post-surgery. Efficacy and safety outcomes will be assessed at 2 weeks, 3, 6, 12, and 24 months post-surgery at regularly scheduled clinic visits. At each time point, participants will complete the PROMs. Physical examination will be performed at 2 weeks, 3, 6, 12, and 24 months post-surgery follow-ups. Shoulder-related adverse events and serious adverse events will also be documented. In cases where the participant does not return to the clinic, study personnel will contact the participant by telephone, text, email, or standard mail. A missed follow-up form should be completed if the participant misses the follow-up visit. The schedule of events (Table 1) details the requirements and procedures for each visit.

**Table 1:** Schedule of events

Assessment/procedures	Visit 1: Enrollment/screening	Visit 2: Surgery	Visit 3: 2 weeks post-surgery (± 7 days)	Visit 4: 3 months post-surgery (± 14 days)	Visit 5: 6 months post-surgery (± 30 days)	Visit 6: 12 months post-surgery (± 30 days)	Visit 7 24 months post-surgery (± 60 days)
Eligibility screening	●						
Informed consent	●						
Collection of demographic, medical history, radiograph	●						
Collection of surgical data		●					
Surgery		●					
WOSI	●		●	●	●	●	●
ASES	●		●	●	●	●	●
Shoulder Activity Scale	●		●	●	●	●	●
EQ-5D	●		●	●	●	●	●
Patient Satisfaction Scale	●		●	●	●	●	●
Physical examination	●		●	●	●	●	●
Radiograph (standard of care, **Latarjet only**)			●				
Follow-up form			●	●	●	●	●
Safety assessment		●	●	●	●	●	●

### Early withdrawal

Participants may decide to withdraw from this trial at any time. If a participant withdraws prior to completing the trial, the study personnel will document the reason for withdrawal and attempt to collect any available outcome data. Participants will not be withdrawn from the study due to lack of adherence to the study protocol (e.g. participant received wrong treatment arm, missed follow-up visits etc.).

### Participant retention

Once a patient is enrolled in the trial, the clinical site will make every reasonable effort to follow the participant for the entire duration of the study period. The past follow-up rates for similar fracture trials performed by the study investigators [36–38] is greater than 90%. Strategies to maintain participant retention include the following: Individuals will be excluded if they are likely to present problems with follow-up (refer to exclusion criteria). At the time of randomization, each participant will provide the name and address of their primary care physician, and the name, address, and phone number of three people at different addresses with whom the participant does not live with who are likely to be aware of the participant’s whereabouts as well as their own address and phone number. The research coordinator will confirm that these numbers are accurate prior to the participant’s discharge from the hospital. Whenever possible, participants will be given information regarding surgical stabilization for recurrent dislocations, potential complications, and benefits and will be encouraged to adhere to follow-up visits and research protocols. The study coordinator will remind participants of upcoming clinic visits. The study coordinator will contact participants no less than once every three months to maintain contact and obtain information about any planned change in residence. Efforts will be made to contact patients to ensure follow-up compliance and that appointment reminders will be conducted by both fracture clinic and study staff to ensure compliance. Because the STABLE study will be a younger population who may be less likely to attend follow-up visits compared to older fracture populations, it is possible that follow-up rates will not be as high as our group’s previous studies, which is a key reason to conduct this pilot trial. We will use the information from this pilot study to refine our participant retention strategies for the definitive trial.

### Trial committees

The Steering Committee will provide guidance and direction; specific responsibilities include reviewing and approving the study protocol and working collaboratively to resolve any challenges that arise during the pilot study. The Steering Committee will be comprised of national and international experts in shoulder and orthopaedic surgery and research methodology. The committee will be blinded throughout the trial. Data and Safety Monitoring Committee (DSMCs) are generally recommended for any controlled trial that will compare rates of mortality or major morbidity. Guidance from the Federal Drug Administration supports that a DSMC is not needed for clinical trials exploring interventions to promote symptom relief (http://www.fda.gov/RegulatoryInformation/Guidances/ucm12069.html). As such, a DSMC will not be used in the pilot trial.

## Statistical plan

### Sample size

The feasibility objectives in our pilot study do not lend themselves to traditional quantitative sample size calculations. If we use CIs limits for the binary outcome of compliance or not at 80% with a CI of 20%, we require 70 patients total. If we account for 15% loss to follow-up, we require a total of 82 patients. Therefore, we propose a sample size of at least 82 participants (41 participants per treatment arm). We have used similar pilot sample sizes to demonstrate feasibility in our previous multi-centre trials [36–39].

### Analysis plan

#### Primary analysis—Feasibility

To meet the feasibility objectives of this pilot randomized controlled trial we have planned descriptive analyses. We will present point estimates of recruitment and feasibility events, including adherence to protocol, follow-up rate at 24 months, and data completeness as proportions with 95% CIs. The pilot study results will be evaluated to identify recruitment issues and data management issues and inform anticipated follow-up rates.

#### Secondary analysis

All outcome analyses will be exploratory only and will adhere to the intention-to-treat principle. For the pilot study, we will perform summary descriptive analyses for recurrence rate and for all clinical outcome measures. This will include measures of central tendency (mean, median) and variance (95% CI, standard deviation, inter-quartile range), or proportions/risks (e.g. RR, odds ratio) where appropriate. Because the pilot trial is not powered to detect differences in clinical outcomes, we will not present *p* values. We will present all point estimates with 95% confidence intervals and graphically overlay minimally important differences (MIDs) to give context.

### Interim analysis

We will not conduct an interim analysis for this pilot trial.

### Data management

#### Case report forms and data transmission

Clinical sites will be provided with the trial case report forms (CRFs) prior to initiation of enrollment. Research personnel at each clinical site will submit the required data, as detailed on the CRFs, to the Methods Centre using the REDCap system. Clinical site personnel will receive a unique login and password for the REDCap system and will be able to view and modify data for participants recruited at their clinical site.

#### Data integrity

The REDCap system uses a variety of mechanisms for checking data at the time of entry including skip logic, range checks, and data type checks. Upon receipt of new data, the personnel at the Methods Centre will query all missing, implausible, or inconsistent data, clinical site personnel will be notified of open queries through regular reports and will be required to respond promptly.

## Ethics and dissemination

This protocol has been reviewed and approved by the Hamilton Integrated Research Ethics Board (HiREB; project number 4942) and will be approved by the local Institutional Review Board (IRB) or REB of each participating clinical site prior to initiation of trial activities at the clinical site. We will seek written informed consent from each study participant.

Any amendments to the study protocol which may affect the conduct of the study, or the potential safety of or benefits to participants (e.g. changes to the study objectives, study design, sample size, or study procedures) will require a formal amendment to the protocol. Any protocol amendments will be approved by the principal investigator and will require approval by the lead site REB and the local REB/IRB at each participating clinical site. Administrative changes (e.g. minor corrections or clarifications that have no effect on the way the study is conducted) will not need to undergo a formal amendment process.

Results from the study will be submitted for publication regardless of whether or not there are statistically significant findings. Every attempt will be made to ensure that the amount of time between completion of data collection and release of study findings is minimized.

## Supplementary Information

**Additional file 1.**

## Data Availability

Not Applicable

## Abbreviations

ASES: American Shoulder and Elbow Society
CEO: Centre for Evidence-Based Orthopaedics
CI: Confidence intervals
CONSORT: Consolidated Standards of Reporting Trials
CRF: Case report forms
CT: Computed tomography
DSMC: Data Safety Monitoring Committee
EQ-5D: EuroQol-5 Dimensions
MID: Minimally important differences
HiREB: Hamilton Integrated Research Ethics Board
IRB: Institutional Review Board
PROMs: Patient-reported outcome measures
REB: Research Ethics Board
REDCap: Research electronic data capture
RR: Risk ratio
SPIRIT: Standard Protocol Items: Recommendations for Interventional Trials
STABLE: Shoulder instability Trial comparing Arthroscopic stabilization Benefits compared with Latarjet procedure Evaluation
WOSI: Western Ontario Shoulder Instability
WSIB: Workplace Safety and Insurance Board
US: Unites States
