# Correction to: A pilot multicenter randomized controlled trial comparing Bankart repair and remplissage with the Latarjet procedure in patients with subcritical bone loss (STABLE): study protocol

**DOI:** 10.1186/s40814-022-01020-4

**Published:** 2022-03-11

**Authors:** Moin Khan, Asheesh Bedi, Ryan Degen, Jon Warner, Mohit Bhandari, on behalf of the STABLE Investigators, Moin Khan, Ryan Degen, Mohit Bhandari, Asheesh Bedi, Jon Warner, Kim Madden, Nazanin Barkhordari, Miriam Garrido Clua, Kelsey Wozny, Jaydeep Moro, Matthew Denkers, Olufemi R. Ayeni, Robert Litchfield, Diane Bryant, Stacey Wanlin, Andrew Firth, Stephanie Horst, Katelyn Inch, Peter Lapner, Katie McIlquham, Montserrat Garcia Portabella, Jorge H. Nuñez, Lledo Batalla, Josep Massons, Patrick Henry, Katrine Milner, Yinmin Ou, Monica Kunz, Alicia Alvares, Saranjan Moganathas, Aarani Chandrasegaram, Etinosa Oliogu, Phumeena Balasuberamaniam, Barbara Gundi, Nithila Sivakumar, Khadija Rashid, Stephanie Lewaniak, Atqiya Fariha, Lavaneyaa Sri, Bashar Alolabi, Carlee Bolton, Xinning “ Tiger” Li, Emily Curry, Dana Michlin, Davide Bardana, Ryan Bicknell, Heather Grant, Fiona Howells, Peter MacDonald, Jason Old, Jarret Woodmass, Sheila Mcrae, Brittany Bruinooge, Derek McLennan, Rahne Magnusson, Timothy Leroux, Tamara Wagner, Michaela Kopka, Mark Heard, Greg Buchko, Sarah Kerslake, Rachel M. Frank, Eric McMarty, Andres Barandiaran, Kelly Leach, Kyle Suess, Bruce Miller, John Grant, Bethany Ruffino, Anand Murthi, Shawanna Jackson, Rodrigo de Marinis Acle, Rodrigo Liendo Verdugo, Catalina Vidal Olate, Michel van den Bekerom, Derek van Deurzen, Sigrid Vorrink, Ydo V. Kleinlugtenbelt, I. F. Kodde, Ellie Landman, Hannie Elskamp-Meijerman, Monique Voskamp, Raul Barco, Alfonso Vaquero, Abdul-ilah Hachem, C. Ventura-Parellada, J. M. Mora Guix, F. Gamez-Baños

**Affiliations:** McMaster University, Mary Grace Wing, Room G807, 50 Charlton Ave E, Hamilton, ON L8N 4A6 Canada

**Correction to: Pilot Feasibility Stud 8, 20 (2022)**

**https://doi.org/10.1186/s40814-022-00987-4**

Following publication of the original article [1], it was noted that due to a typesetting error the list of the STABLE Investigators are incomplete.

The complete list of investigators are listed below:

Moin Khan

Ryan Degen

Mohit Bhandari

Asheesh Bedi

Jon Warner

Kim Madden

Nazanin Barkhordari

Miriam Garrido Clua

Kelsey Wozny

Jaydeep Moro

Matthew Denkers

Olufemi R. Ayeni

Robert Litchfield

Diane Bryant

Stacey Wanlin

Andrew Firth

Stephanie Horst

Katelyn Inch

Peter Lapner

Katie McIlquham

Montserrat Garcia Portabella

Jorge H. Nuñez

Lledo Batalla

Josep Massons

Patrick Henry

Katrine Milner

Yinmin Ou

Monica Kunz

Alicia Alvares

Saranjan Moganathas

Aarani Chandrasegaram

Etinosa Oliogu

Phumeena Balasuberamaniam

Barbara Gundi

Nithila Sivakumar

Khadija Rashid

Stephanie Lewaniak

Atqiya Fariha

Lavaneyaa Sri

Bashar Alolabi

Carlee Bolton

Xinning “Tiger” Li

Emily Curry

Dana Michlin

Davide Bardana

Ryan Bicknell

Heather Grant

Fiona Howells

Peter MacDonald

Jason Old

Jarret Woodmass

Sheila Mcrae

Brittany Bruinooge

Derek McLennan

Rahne Magnusson

Timothy Leroux

Tamara Wagner

Michaela Kopka

Mark Heard

Greg Buchko

Sarah Kerslake

Rachel M. Frank

Eric McMarty

Andres Barandiaran

Kelly Leach

Kyle Suess

Bruce Miller

John Grant

Bethany Ruffino

Anand Murthi

Shawanna Jackson

Rodrigo de Marinis Acle

Rodrigo Liendo Verdugo

Catalina Vidal Olate

Michel van den Bekerom

Derek van Deurzen

Sigrid Vorrink

Ydo V. Kleinlugtenbelt

I.F. Kodde

Ellie Landman

Hannie Elskamp-Meijerman

Monique Voskamp

Raul Barco

Alfonso Vaquero

Abdul-ilah Hachem

Ventura-Parellada C

Mora Guix JM

Gamez-Baños F

The original article [1] has been corrected.
