# Supplementary material for: A pilot multicenter randomized controlled trial comparing Bankart repair and remplissage with the Latarjet procedure in patients with subcritical bone loss (STABLE): study protocol
Source: Pilot Feasibility Stud. 2022 Jan 31;8:20. doi: 10.1186/s40814-022-00987-4 (PMC8802453; doi:10.1186/s40814-022-00987-4)
Supplement: Supplementary file 1 — Additional file 1. [file 40814_2022_987_MOESM1_ESM.docx]

**Appendix A – Standardized Rehabilitation Protocol**

**Phase I**: Immediate postop phase (first 5-7 days after surgery, prior to starting PT)

- Sling always except for when doing therapy;
- Pendulum exercises, Supine external rotation, Supine passive arm elevation, Scapular retraction, Shoulder shrug.

**Phase II**: Intermediate postop phase (5-7 days post-surgery – 2 weeks)

- Begin formal physiotherapy;
- Exercises focus on stretching and passive motion: Supine external rotation (limit to 0 degrees ER), Supine forward arm elevation, Ball squeeze exercise (squeeze rubber ball in hands for 5-7 seconds), Scapular retraction.

**Phase III**: Initiate motion phase (weeks 2 to 5)

- *Stretching/PROM:* Pendulum exercises, Supine external rotation (limit to 30 degrees), Supine passive arm elevation, Behind the back internal rotation (start at 3 weeks postop);
- *Isometrics:* Internal and external rotation at neutral, Prone row, Prone extension (do not extend past hip), Side lying external rotation, Rhythmic stabilization and proprioceptive exercises with therapist.

**Phase IV**: Active motion phase (5-8 weeks after surgery)

- *Stretching/AROM:* Pendulum exercises, Supine ER (limit to 45 degrees), Standing ER (limit to 45 degrees), Supine passive arm elevation, Seated-standing forward arm elevation, Behind the back IR;
- *Strengthening/Theraband*: External rotation, Internal rotation, Prone row, Prone horizontal abduction “T’s”, Prone extension, standing scaptation, Side-lying ER, Rhythmic stabilization and proprioception exercises with therapist.

**Phase V**: Strengthening phase (8-12 weeks after surgery)

- *Stretching/PROM:* Pendulums, Standing ER / door / wall slide stretch, Hands behind the head stretch (starting at 9 weeks), Behind the back IR, Supine cross body stretch, Side lying IR;
- *Strengthening/Theraband:* External rotation, Internal rotation, Standing forward punch, Shoulder shrug, Dynamic hug, Wall “W’s”, Seated row;
- *Strengthening/Dynamic:* Side lying ER, Prone horizontal abduction “T’s”, Prone scaptation “Y’s”, Prone row, Prone extension, Standing scaptation “full can” exercises, Rhythmic stabilization and proprioception exercises with therapist.

**Phase VI**: Advanced strengthening phase (12 weeks and beyond)

- *Stretching/PROM:* Continue phase V exercises, External rotation at 90 degrees’ abduction stretch;
- *Strengthening/Theraband:* Continue phase V exercises, External rotation at 90°, Internal rotation at 90°, Standing ‘T’s, Diagonal up, Diagonal down.
- *Strengthening/Dynamic:* Continue phase V exercises, Prone ER at 90 degrees’ abduction “U’s”, Biceps curls, resisted forearm supination/pronation, resisted wrist flexion/extension, PNF manual resistance with therapist, Push up progression
